# Improvement of cell suspension cultures of transformed and untransformed *Carica papaya* cell lines, towards the development of an antiparasitic product against the gastrointestinal nematode *Haemonchus contortus*

**DOI:** 10.3389/fcimb.2022.958741

**Published:** 2022-09-08

**Authors:** Anabel Ortiz Caltempa, Marisela Hernández, Ana Lilia Pérez, Liliana Aguilar, Cynthia Guzmán, Dolores Adriana Ayón-Núñez, Gladis Fragoso, Raúl J. Bobes, Maria Eugenia López, Edda Sciutto, María Luisa Villareal

**Affiliations:** ^1^ Centro de Investigación en Biotecnología, Universidad Autónoma del Estado de Morelos, Cuernavaca, Morelos, Mexico; ^2^ Laboratorio de Inmunología, Departamento de Inmunología, Instituto de Investigaciones Biomédicas, Universidad Nacional Autónoma de México, Ciudad de México, Mexico; ^3^ Centro de Investigación Disciplinaria en Salud Animal e Inocuidad, Instituto Nacional de Investigaciones Forestales, Agrícolas y Pecuarias, Jiutepec, Morelos, Mexico

**Keywords:** *Carica papaya*, transformed callus, transformed cell suspension, nematocidal activity, *Haemonchus contortus*

## Abstract

Parasitic diseases have a major impact on human and animal health worldwide. Despite the availability of effective anti-parasitic drugs, their excessive and uncontrolled use has promoted the emergence of drug resistance, severely affecting ecosystems and human health. Thus, developing environmentally friendly antiparasitic treatments is urgently needed. *Carica papaya* has shown promising effects against infectious diseases. *C. papaya* embryogenic calluses were genetically modified by our research team to insert immunogenic peptides with the goal of developing an oral anti-cysticercosis vaccine. Among these callus cell lines, one labeled as CF-23, which expresses the KETc7 immunogenic peptide, induced the highest protection levels against experimental cysticercosis. In the process of designing a natural antiparasitic product based on *C. papaya* that simultaneously induced immunity against cysticercosis, both transformed (SF-23) and untransformed (SF-WT) suspension cultures were produced and optimized. Our results showed a better duplication time (td) for SF-23 (6.9 days) than SF-WT (13.02 days); thus, the SF-23 line was selected for scale-up in a 2-L airlift bioreactor, reaching a td of 4.4 days. This is the first time that a transgenic line of *C. papaya* has been grown in an airlift bioreactor, highlighting its potential for scale-up cultivation in this type of reactor. Considering the previously reported nematocidal activity of *C. papaya* tissues, their activity against the nematode *Haemonchus contortus* of aqueous extracts of SF-WT and SF-23 was explored in this study, with promising results. The information herein reported will allow us to continue the cultivation of the transgenic cell suspension line of *C. papaya* under reproducible conditions, to develop a new anti-parasitic product.

## Introduction

Parasitic diseases are known to have a serious impact on human and animal health, compromising the survival and reproduction of both wildlife and livestock. A recent trend in vaccine design and development is based on the use of transgenic plants to produce orally administered vaccines. Among other advantages, the oral route could stimulate mucosal immunity, the first line of defense. Moreover, due to the presence of cell walls, endomembranes, and biopolymers, plant cells have the potential to encapsulate antigens, protecting them from digestion and allowing them to elicit systemic immunity (Rosales and Salazar, 2014; Chan and Daniell, 2015).

A peptide-based vaccine was developed in the last two decades to interrupt the transmission of swine cysticercosis (Sciutto et al., 2007). In its first iteration, the vaccine consisted of three synthetic peptides named KETc1, KETc7, and KETc12, which proved to induce high protection levels in pigs exposed to natural challenges of *Taenia solium* cysticerci (Huerta et al., 2001). In a second version of the vaccine, recombinant peptides were expressed in filamentous phages, with similar encouraging results (Morales et al., 2011). However, both versions were injectable vaccines, whose production involved high costs, and whose application in backyard pigs (their intended target) proved to be logistically difficult. To overcome both issues, KETc1, KETc7, and KET12 were expressed in *C. papaya* embryogenic calluses to produce an oral vaccine (Hernández et al., 2007).

*C. papaya* is an herbaceous plant, native to Mesoamerica (SNICS, 2018). Its fruit is high in antioxidants, vitamin B, and elements like potassium and magnesium. It also contains an enzyme called papain, which aids digestion and is used to treat gastritis, colitis, and chronic constipation. *C. papaya* has also been shown to have anthelmintic properties due to its content of cysteine proteinases such as chymopapain, papain, caricain, and glycyl endopeptidase (Stepek et al., 2004; Hounzangbe-Adote et al., 2005; Njom et al., 2021); all these compounds are concentrated in the fruit latex and seeds (Luyando, 2012).

Genetic constructs encoding the peptides KETc1, KETc7, and KETc12 were used to transform embryogenic callus of *C. papaya* by biolistics. Transgenic tissues were selected by co-transforming the pUI235-5.1 vector constructs with the pWRG1515 (carrot and papaya) and pBARGUS (maize and sorghum) vectors; the presence of the GUS reporter gene, as well as hygromycin and herbicide resistance genes, was also induced. Selected embryogenic transgenic callus were propagated and sub-cultured on nutrient medium supplemented with hygromycin and kanamycin. The clones were molecularly characterized, verifying the production of KETc1, KETc7, and KETc12 (Hernández et al., 2007).

Transgenic lines expressing KETc1, KETc7, and KETc12 protected mice from *T. crassiceps* challenges by 50%, 63%, and 80%, respectively (Fragoso et al., 2017). The protective capacity induced by immunization with extracts from transgenic cell lines could be due to the synergistic effect of papaya extract per se and the response induced by the recombinant peptides (Fragoso et al., 2017). Thus, a stable platform was generated to produce an oral vaccine against cysticercosis in papaya calluses, expressing the peptides KETc1, KETc7, and KETc12 (Hernández et al., 2007; Fragoso et al., 2017).

Given the potential of transgenic plants for vaccine production, this work is aimed at optimizing the culture conditions of transgenic callus and callus-derived cell lines in suspension. Thus, callus lines expressing the three peptides that form the vaccine were developed and tested for their suitability for biomass production in suspension culture. Due to its friable consistency, the callus line CF-23 was best adapted for cell suspension cultures. Interestingly, this cell line expresses KETc7, which is part of K7, a larger antigenic peptide whose properties had been characterized molecularly and immunologically (Bobes et al., 2017). Furthermore, KETc7 contains an immunomodulatory epitope called GK-1 (Montero et al., 2020), which has shown the highest protective capacity against murine cysticercosis (Sciutto et al., 2013).

The anthelmintic effect of papaya, in particular, due to its content of cysteine proteases, has been evaluated on *Trichuris muris* (Stepek et al., 2006), *Protospirura muricola* (Stepek et al., 2007), *T. suis, Ascaris lumbricoides*, and *T. trichiura*, as well as hookworms (Levecke et al., 2014), *Hymenolepis diminuta* (Mansur et al., 2016), and *Haemochus contortus* (Buttle et al., 2011).

*H. contortus* larvae are frequently found in the abomasum of sheep, and their hematophagous activity can cause mucosal hemorrhages, along with gastritis, anemia, and other complications, even causing death in heavily parasitized animals (Acevedo-Ramírez et al., 2019). Therefore, the nematocidal capacity of papaya callus on L_4_ *H. contortus* larvae was evaluated in this work. The differential protein expression profile of wild-type and KETc7-expressing calluses was also determined.

## Materials and methods

### Callus cultures

An embryogenic transgenic *C. papaya* callus line expressing the recombinant peptide KETc7 (CF-23) was previously obtained by transforming *C. papaya* tissues by biolistics (Hernández et al., 2007). Transformed (CF-23) and non-transformed (CF-WT) *C. papaya* callus lines were selected and maintained in B5 medium (Gamborg et al., 1976) supplemented with 30 g/L sucrose, 3 g/L polyvinylpyrrolidone, and 1.5 g/L phytagel); a combination of phytoregulators, 2.0 mg/L 2,4-dichlorophenoxyacetic acid (2,4-D), plus 2.0 mg/L kinetin (KIN), was added and evaluated. The pH was adjusted to 5.7 ± 0.2, and the medium was autoclaved at 121°C for 15 min. The cultures were maintained at 25 ± 2°C either under constant light (50 µmol m^−2^ s^−1^, provided by fluorescent lamps), under photoperiod (16 h light and 8 h dark), or in the dark. All lines were sub-cultured every 20 days under the same conditions until friable calluses were established.

The CF-23 and CF-WT calluses used in this study to produce SF-23 and SF-WT, respectively, were kindly donated by Dr. Edda Sciutto, Department of Immunology, Instituto de Investigaciones Biomédicas, UNAM, Mexico City, Mexico. Herein, the authors declare that all plant material was cultured in accordance with all institutional, national, and international guidelines/regulations/legislation.

### Identification of the KETc7 transgene

The presence of the KETc7 transgene in the CF-23 line was detected by PCR using genomic DNA isolated with the DNeasy Plant Mini Kit (Qiagen, Valencia, CA). The following primers were used: forward LHE-1700 (5∗ GGA TGA CGC ACA ATC CCA CTA T 3∗) from −84 bp of the CAMV 35-35S promoter and the A05 reverse primer (5∗ CTA AAG ATT CTT CTT ATC TTC TGG TTC CAT 3∗). The PCR reaction was performed with Platinum Taq Polymerase (Invitrogen, Carisband, CA). The amplified products were analyzed on 2% (w/v) agarose gels (Hernández et al., 2007). GNU Image Manipulation Program v.2.10.14 (https://www.gimp.org) was used to adjust brightness and contrast, and to crop the gel image.

### Differential protein expression profile

#### Protein extracts

Protein extracts from CF-23 and CF-WT were prepared using a 10% trichloroacetic acid solution diluted in acetone. For maceration, papaya embryogenic calluses were placed in 15-ml tubes with extraction solution. The tubes were sonicated with 10-s pulses and allowed to cool on ice for 1 min. The macerate was incubated on ice for 2 min and then centrifuged at 12,000 rpm for 20 min at 4°C, discarding the supernatant. Cold acetone was then added, vortexed, and centrifuged at 12,000 rpm for 20 min at 4°C, discarding the supernatant. Then, cold methanol was added, vortexed, and centrifuged again at 12,000 rpm for 20 min at 4°C, discarding the supernatant. Protein content was quantified using a 2-D Quant Kit (Amersham Bios-Sciences, Little Chalfont, UK), the samples were frozen at −70°C until used.

### Two-dimensional electrophoresis on SDS polyacrylamide gels

Two-dimensional electrophoresis on SDS polyacrylamide gels (2D-SDS-PAGE) were prepared to characterize extracts from the SF-23 and SF-WT lines. Briefly, 100 μg of protein extract were resuspended in rehydration buffer (7 M urea, 2 M thiourea, 4% CHAPS, 0.86 M DTT, 2% ampholine, and 0.1% bromophenol blue) to a final volume of 125 μl. The samples were applied on immobilized pH 3–10 linear gradient strips (7 cm, Bio-Rad) for 16 h. The strips were covered with 2 ml of DryStripCover Fluid mineral oil to prevent sample evaporation. Isoelectric focusing was performed on a Protean IEF Cell kit. Focusing started at 300 V for 1 h; then, voltage was increased to 1000 V for 30 min, and finally to 5000 V for 2 h in an Ettan IPG-phor III electrophoresis unit (GE Healthcare). For the second dimension, the strips were equilibrated for 15 min in sample buffer (6 M urea, 50 mM Tris-Cl pH 8.8, 30% glycerol, 2% SDS, and 0.1% bromophenol blue) with 1% DTT. The strips were then overlaid onto a 12% SDS-PAGE. Polyacrylamide gels were stained with Instant Blue stain (UltrafastProteinStain ISB1L, Sigma).

### Cell suspension cultures

For cell suspension cultures, 10 g fresh-weight (FW) of friable calluses of the lines CF-23 and CF-WT were inoculated into 250 ml Erlenmeyer baffled flasks containing 100 ml of B5 medium + 2,4-D:KIN, 2:2 mg/L, without phytagel. The cultures were grown at 25 ± 2°C in a rotatory shaker at 110 rpm in the dark. Subcultures were taken every 2 weeks until fine cells were obtained, and these were used as inoculum for shaker flasks. Suspensions of the SF-23 and SF-WT cell lines were cultured and maintained in B5 liquid medium added with 2,4-D:KIN, 2:2 mg/L under the conditions described above. For kinetic studies, the cultures were grown for 42 days and sampled at 3-day intervals, recording fresh weight (FW), dry weight (DW), cell viability, pH, and carbohydrate consumption. To evaluate FW, each flask was filtered through medium-pore filter paper disks. The samples were freeze-dried and cell dry weight was recorded. Cell viability was measured by the fluorescein diacetate (FDA) method. Carbohydrate (sucrose, glucose, and fructose) consumption was estimated by determining their concentrations in culture medium by a high-performance liquid chromatography (HPLC) method [Waters 7117 with autosampler; 5-µm, 4.6 mm × 150 mm UG80 amino column, mobile phase (80:20), acetonitrile:HPLC-grade water, 1 ml/min at 25°C].

### Cell suspension cultures in airlift bioreactor

To scale-up suspension cultures, 2-L custom-made airlift bioreactors, 52-cm height, 7-cm diameter, 27-cm draft tube height, 2.7-cm inner draft tube diameter, and 2.0-cm bottom clearance were used. Air is sprayed at the bottom of the draft, generating an internal loop with upcomer in the draft and downcomer in the ring (Caspeta et al., 2005). The empty airlift bioreactor was sterilized and filled with autoclaved B5 medium (1.8 L, supplemented with 30 g/L sucrose and 2:2 mg/L 2,4-D:KIN. The suspension cell line SF-23 was selected for scale-up because of to their good growth in flask cultures. A 15-day-old SF-23 cultures grown in 1-L Erlenmeyer flasks containing 500 ml of B5 medium were used to produce an inoculum of 10% FW (v/v). The bioreactor was incubated at 25 ± 2°C under continuous light (white light flux density of 50 μmol/m^2^/s) for 33 days. The bioreactor was operated at 0.1 vvm for 15 days and then at 0.8 vvm, until the end of the culture period. These conditions allowed for an adequate mixing of the cell suspension throughout the culture period. Antifoam (Dow Corning FG-10) was added as required as a 0.5-ml injection (0.1% v/v). The culture was sampled every 3 days to measure biomass, pH, and sugar content.

### Statistical analysis of culture kinetic parameters

Culture data are reported as mean ± standard deviation of three independent replicates and were analyzed by ANOVA and a Tukey test. Using biomass data (mean of three experiments) from cell cultures in shake flasks and bioreactors, growth curves were plotted, and the specific growth rate (µ) was calculated from the slope of the straight line obtained by plotting ln *x vs*. time *t* during the exponential growth phase. Doubling time (td) was determined with the equation *td* = ln 2/μ.

### Nematocidal activity against *H. contortus* larvae (L_4_)

A 4-month-old male sheep was used as egg donor. The sheep was previously dewormed and then it was orally infected with 350 infective *H. contortus* larvae (L_3_)/kg of live weight. After a 21-day pre-patent period, fecal samples were collected directly from the rectum and disaggregated in plastic containers; tap water was added to maintain humidity. The fecal cultures were covered and incubated at room temperature (25–30°C), shaking daily to oxygenate. After 7 days of incubation, the larvae were recovered by the Baermann funnel technique (Liébano et al., 2011). Fourth-stage *H. contortus* larvae were obtained from infective sheath-less larvae and recovered in a 50-ml tube with 20 ml of sterile 1 M PBS, pH 7.4. Larvae were incubated for 2 h at 37°C and 50 rpm and placed in culture boxes containing Hanks’ medium, supplemented with 400 μl of amphotericin B, 200 μl of antibiotic solution, and 10 μl of sheep blood. Larvae were cultured at 37 °C; under 5% CO_2_. Aeration was provided, and medium was changed every three days until L_4_ larvae were obtained in 14 days (González-Cortázar et al., 2021).

### *In vitro* bioassays

For a preliminary evaluation of the bioactivity of the *C. papaya* SF-23 and SF-WT cell lines against L_4_ *H. contortus* larvae, four SF-WT samples (0, 12, 30, or 42 days of growth) and 15 SF-23 samples (0, 3, 6, 9, 12, 15, 18, 21, 24, 27, 30, 33, 36, 39, or 42 days of growth) were used. Each extract was diluted to a concentration of 1 mg/ml in PBS, pH 7.4. One hundred L_4_ *H. contortus* larvae per well were placed in a microtiter plate containing the SF-WT or SF-23 extracts. PBS, pH 7.4, and Hanks’ medium were used as negative controls. Commercial Ivermectin (SIGMA 70288-86-7) analytical grade at a concentration of 5 mg/ml was used as a positive control. The plates were incubated for 72 h at 37 ± 1°C. Larval viability was determined with a Digital/Digi 2, 15000 Labomell optical microscope (10X), and the percentage of mortality was calculated. Those extracts with the higher effect against L_4_ *H. contortus* larvae were selected for further evaluation.

### Ethical standards

This study was approved by the National Institute of Agricultural and Livestock Forestry Research, at Jiutepec, Morelos, México. It was conducted in accordance with the approved techniques for animal use and care in Mexican Federal Guidelines (NOM-051-ZOO-1995, http://www.senasica.gob.mx: humanitarian care of animals during mobilization; NOM-062-ZOO-1995: technical specifications for the care and use of laboratory animals-livestock farms; farms; centers of production, reproduction, and breeding). Additionally, the INIFAP certified that all the procedures performed for the generation of the results herein reported were in accordance with the ethical standards and the Federal Laws and regulations cited above and conducted according to the ARRIVE guidelines.

### Statistical analysis

All results are reported as mean (μ) ± SD standard deviation of three independent replicates. The effect on L_4_ *H. contortus* larval mortality was determined with the following formula:


$$\%\;Larval\;mortality=\frac{\mu \;negative\;control-\mu \;treatment\;group}{100-\mu \;negative\;control}*100$$

Data were tested for normality and analyzed by analysis of variance using a completely random design in the SAS (V9) program (SAS Institute, 1998). Additionally, the Tukey statistical test was used for the comparison of means.

## Results

### Callus cultures

**Figure 1:**
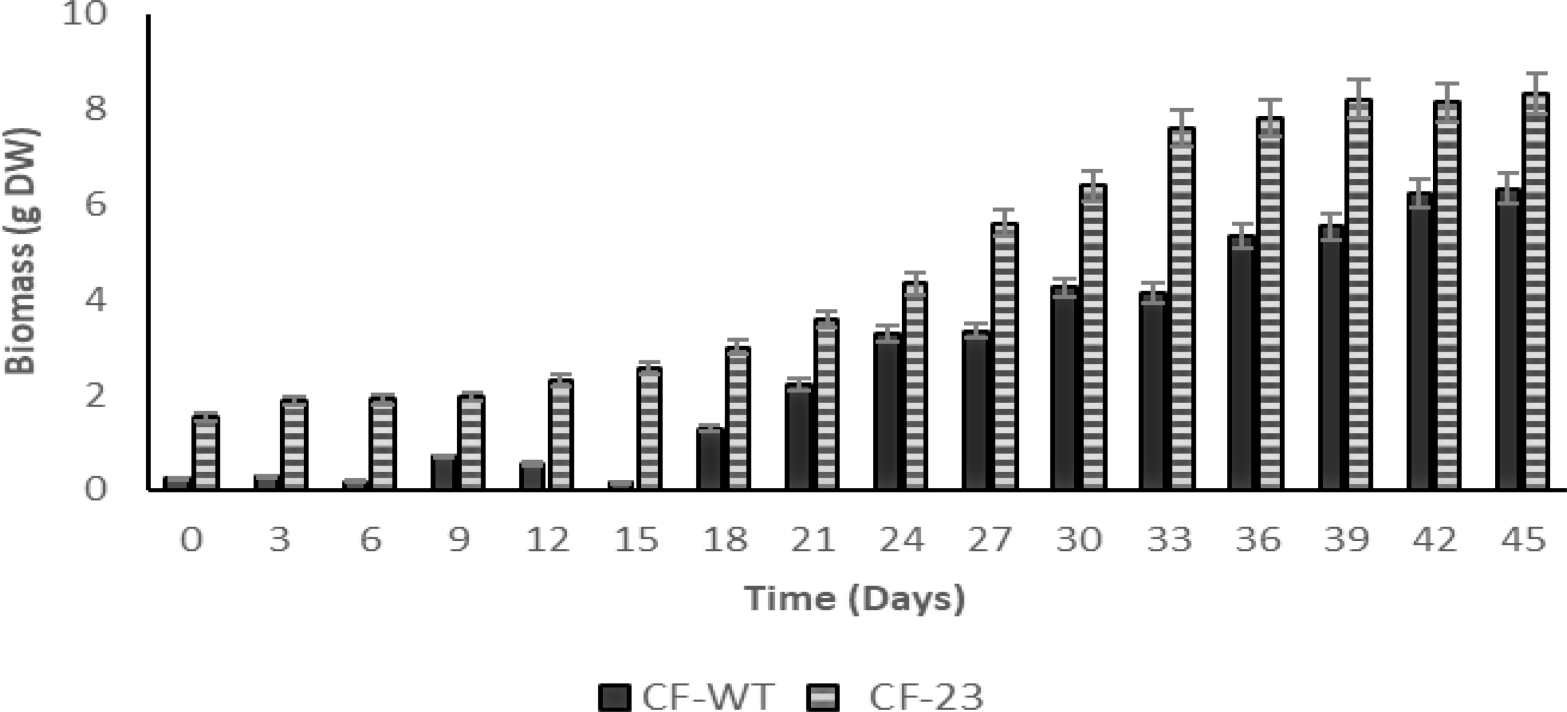
Growth of *C. papaya* CF-23 and CF-WT callus lines in B5 medium + 2,4 D:KIN, 2:2 mg/L in the dark. Data are expressed as mean ± SD of three independent experiments. Significant differences between CF-WT and CF-23 were observed, *P* = 0.02938047, α = 0.05 for ANOVA and Tukey’s test (*P* < 0.05).

To assess the growth of transgenic (CF-23) and wild-type (CF-WT) calluses, both calluses’ cell lines were cultured for 45 days, recording biomass DW every 3 days. As shown in **Figure 1**, the CF-23 cell line exhibited a better growth on solid B5 medium + 2,4-D:KIN, 2:2 mg/L in the dark than CF-WT. An optimal growth was observed with the CF-23 line after 45 days of culture, with a biomass of 8.33 g DW, while maximum biomass yield for CF-WT was 6.32 g DW on day 45 **(** **Figure 1**).

### Detection of the KETc7 Transgene in *C. papaya* CF-23 cultures

**Figure 2:**
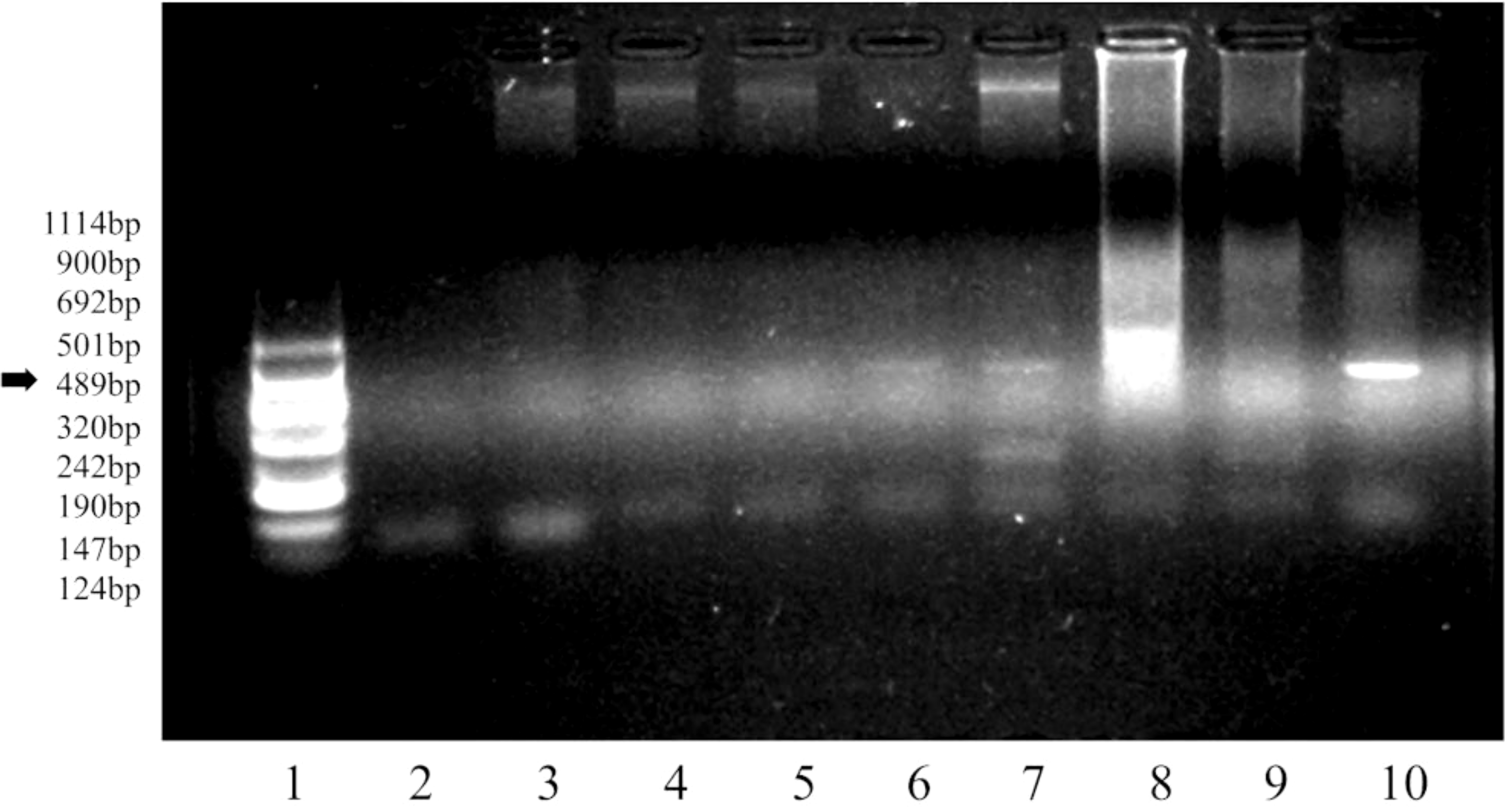
Detection of the KETc7 transgene by PCR. Agarose 1.5% gel in TBE 1X buffer, run for 1.30 h at 110 volts. Lane 1, molecular weight VIII (Roche); 2, primers A04/A05; 3, CF-WT line 3186; 4, CF-WT_0_; 5, CF-WT_9_; 6, CF-23_0_; 7, CF-23_9_; 8, genomic *T. crassiceps* DNA; 9, genomic *T. solium* cysticercus DNA; 10, KETc7/pUI235 genetic construct. The arrow indicates the expected molecular weight of the amplified DNA fragment.

Once the *C. papaya* CF-23 transgenic line callus was formed, the presence of the KETc7 sequence was confirmed by polymerase chain reaction (PCR) (**Figure 2**). The KETc7 insert in CF-23 cells is observable in lanes 6 and 7 at days 0 and 9.

### Protein expression profiles in CF-23 and CF-WT

**Figure 3:**
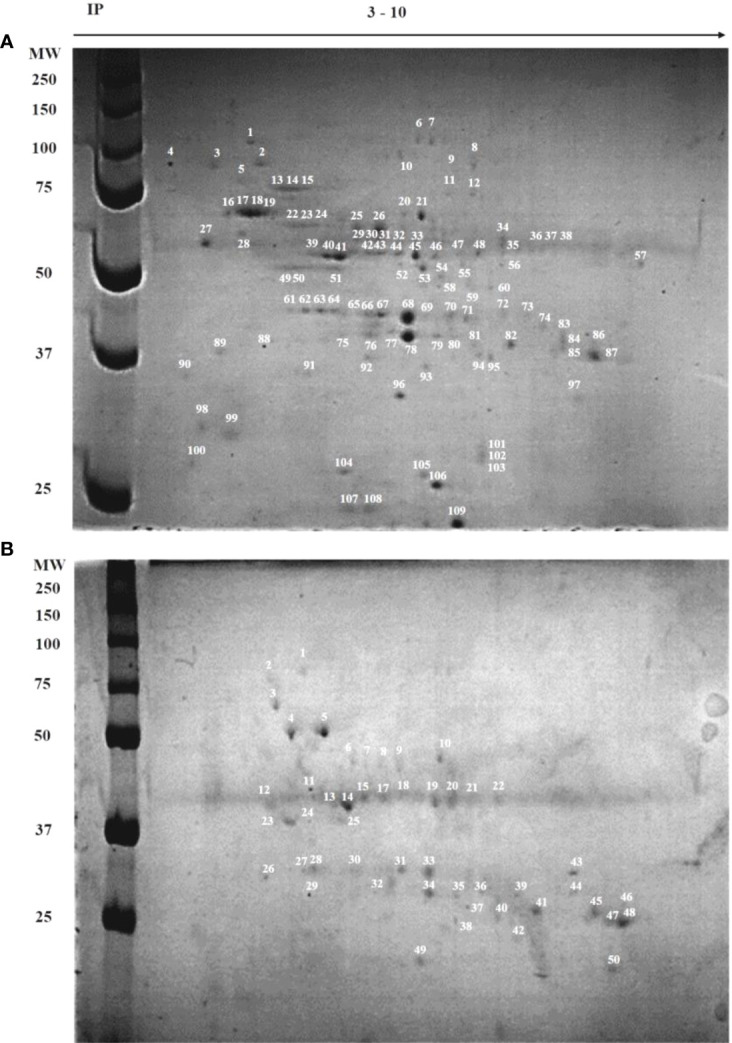
Two-dimensional electrophoresis (IP: 3–10) of wild-type and transgenic *C. papaya* callus cell lines. **(A)** CF-WT line; **(B)** CF-23 line. The proteins labeled by numbers were identified (supporting information, **Supplementary Table 1**). Left lanes show molecular weight markers (MW), isoelectric point (IP).

As a first approach to compare the protein profiles of the CF-23 and CF-WT callus cells, 2D-SDS-PAGE maps of total proteins were obtained. In total, 109 spots were counted in the CF-WT cell line (MW 24–150 kDa; IP 4–7), whereas 50 spots were counted in CF-23 (MW 23–85 kDa; IP 5-7) (**Figure 3**). Most of the spots in wild-type cells were in the region 40–140 kDa, IP 5–7; on the other hand, most of the spots in CF-23 were in the 25–37 kDa region. Some protein spots were shared by both cell lines, including spots number 14 and 75 in WT cells, which matched spots number 2, 15, and 20 in CF-23; spot number 20 in CF-23 matched spots 76, 78, 79, and 80 in WT (**Supplementary Table 1**).

### Cell suspension cultures

**Figure 4:**
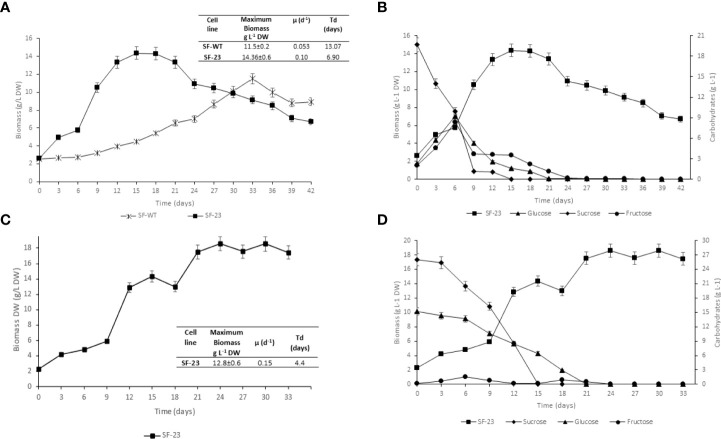
Characterization of *C. papaya* suspension cell lines. **(A)** Biomass growth of *C. papaya* SF-WT and SF-23 cell lines in Erlenmeyer shake flasks (100 ml). Kinetic parameters of *C. papaya* cell suspension batch cultures in flasks (100 ml) are summarized in the Table. Growth rates in the SF-WT and SF-23 cell lines were statistically different according to Tukey’s test (*P*< 0.05). **(B)** Carbohydrate consumption (sucrose, glucose, and fructose) of *C. papaya* SF-23 cell line in Erlenmeyer shake flasks (100 ml). **(C)** Biomass growth of *C. papaya* SF-23 cell line during culturing in airlift bioreactor. Kinetic parameters *C. papaya* SF-23 suspension cell line in airlift bioreactor are shown in the Table. **(D)** Carbohydrate consumption (sucrose, glucose, and fructose) for *C. papaya* SF-23 cell line in airlift bioreactor. Data are reported as mean ± SD of one experiment with three replicates.

*C. papaya* suspension cell lines SF-23 and SF-WT were grown in batch culture for 42 days in flasks. Growth kinetics in Erlenmeyer shake flask culture (100 ml) is shown in **Figure 4A**. The kinetic parameters (maximum biomass, growth rate, and td) are summarized in the table in **Figure 4**. The SF-23 cell line showed better cell growth, reaching 14.36 g/L at day 15, compared with the SF-WT line, which yielded a maximum biomass of 11.5 g/L at day 33. The td for the SF-23 line was less than half of that found for SF-WT (*P*< 0.05).

Since the transgenic cell line SF-23 showed good kinetic parameters, its carbohydrate metabolism was characterized, recording total sugar consumption. As shown in **Figure 4B**, sucrose was hydrolyzed completely to glucose and fructose by day 15; both sugars were depleted by day 21 of culture.

In view of the favorable kinetic parameters in shake flasks, the growth of SF-23 was scaled-up in an airlift bioreactor. Kinetic data for batch cultivation of the *C. papaya* SF-23 cell line in an airlift bioreactor were as follows: exponential growth phase lasted from day 6 to day 15, followed by a stationary phase, achieving a maximum growth of 12.8 g/L by day 12, a 6.38-fold increase over the initial inoculum (**Figure 4C**). Due to foaming during the exponential growth phase, an anti-foaming agent (0.1% v/v) was added. The specific growth rate (µ) for SF-23 was 0.15, and doubling time (td) was 4.4 days (Table in Figure). The biomass yield (Y_X/S_) in the bioreactor at day 12 was 0.0661 g. The carbohydrate consumption pattern in the bioreactor was comparable to that found in flasks (**Figure 4D**).

### Nematocidal activity against *H. contortus*

**Figure 5:**
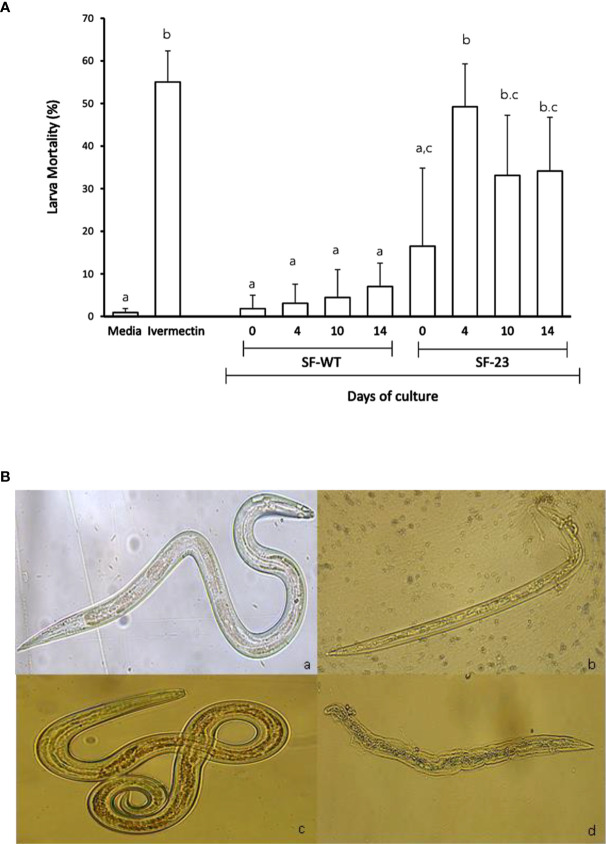
Larval damage induced after 72 h of *in vitro* culture. **(A)** Mortality percentage (mean ± SD) of L_4_ *H. contortus* larvae exposed to 1 mg/ml of *C. papaya* SF-WT and SF-23 cell line extracts sampled at different times of cell suspension culture (0, 4, 10, and 14 days). Larvae cultured in medium alone were included as a negative control, and Ivermectin was used as a positive control. Letters indicate significantly differences (*P* < 0.05) in Tukey’s test. **(B)** Representative images of L_4_ *H. contortus* larvae treated with (a) Hanks’ medium (4X), (b) Ivermectin (10X), (c) SF-WT cell line (14 days) (20X), and (d) SF-23 cell line (14 days) (20X).

The effect of *C. papaya* SF-WT and SF-23 cell lines on the viability of L_4_ *H. contortus* larvae after 72 h of culture is shown in **Figure 5A**. As shown, SF-23 induced greater damage in larvae than SF-WT. The highest mortality rate was induced by the SF-23 transgenic line sampled from day 4 of culture. In fact, it caused a similar effect on larval mortality to that due to Ivermectin, close to 50%.

Representative images of morphological alterations on L_4_ *H. contortus* larvae cultured with *C. papaya* SF-23 and SF-WT cell line extracts, as well as negative (Hanks’ medium) and positive (Ivermectin) controls, are shown in **Figure 5B**. No damage was observed in the negative control group (a). Ivermectin caused death of L_4_ *H. contortus* larvae (b). No damage was observed in larvae cultured with SF-WT extracts (c). Clear damage was found in larvae cultured with the extract from the SF-23 transgenic line sampled at day 14 of culture. As shown, larvae presented a slight dehydration and damage in the middle and posterior regions, as well as disintegration of some internal organs. It should be noted when exposed to SF-23 extract, the L_4_ larvae had already developed an oral cavity and were feeding on blood.

## Discussion

This study is based on a previous work, where *in vitro* growing conditions were established to produce two friable-looking, *C. papaya* cell line calluses: the wild-type line CF-WT and the transgenic line CF-23. One goal of this work was to produce suspension cells lines (SF-WT and SF-23) suitable to be scaled-up at larger volumes and generate higher amounts of bioactive biomass. The non-transformed wild-type line and the transgenic line that induced the highest protection levels against cysticercosis (Fragoso et al., 2017) were selected. Considering previous reports on nematocidal properties of papaya (Stepek et al., 2004), the biomasses obtained were tested *in vitro* against the nematode *H. contortus.*

Both wild-type and transgenic *C. papaya* cell-line calluses showed good growth rates in B5 culture medium. The CF-23 line showed the best cell growth parameters, and the presence of the insert in its genome was corroborated.

In suspension culture, the growth of *C. papaya* SF-WT and SF-23 cell lines showed a 6-day lag phase at the flask level (**Figure 4A**). This behavior is consistent with previous reports on adaptation to the nutritional conditions of the medium (Shigeta et al., 1996). However, the duration of this period may vary depending on the species; for instance, the lag phase was 9 days for *Uncaria tomentosa* (Sánchez-Calvo and Alvarenga-Venutolo, 2015). It is noteworthy that both of our cell lines were kept under the same experimental conditions (culture medium, agitation, and darkness).

The SF-WT line had a specific growth rate of 0.053/d, and a td of 13.07 days, while the specific growth rate for SF-23 was 0.10/d; a shorter td, of 6.9 days, was also found for the transgenic cell line. These are favorable parameters for cell multiplication, in line with previous reports for other transgenic plant cultures. For example, for potato (*Solanum tuberosum*) cell suspension cultures used as a platform to produce recombinant proteins, μ was 0.12/d and td was 5.75 days (Nova-López et al., 2017). For carrot (*Daucus carota*) cell suspensions, μ was 0.06/d, and td was 10 days (Campos, 2019); for tobacco (*Nicotiana benthamiana*) cells, μ was 0.11/d and td was 6.1 days (Sukenik et al., 2018).

The specific growth rate of the SF-23 transgenic line doubled that observed in the SF-WT line. At day 33, the SF-WT line reached a maximum biomass concentration of 11.5 g/L, for a yield of 0.328 g/L; at day 42, a final biomass concentration of 8.94 g/L was reached. On the other hand, the SF-23 transgenic line of C. papaya reached a maximum biomass concentration of 14.36 g/L at day 15, for a yield of 0.391 g/L. Interestingly, the decline of this line was also faster; after 42 days of growth, a final biomass concentration of 6.687 g/L was obtained.

In the death phase, cell aggregates have been observed to decrease in size; this is attributed to the activation of enzymes that degrade cell wall polysaccharides (Jiménez, 2005; Rodríguez-Vivasa et al., 2017). The kinetics of sugar consumption by the *C. papaya* line in flasks indicates that sucrose was hydrolyzed at day 15, while preferential consumption of glucose was observed until day 21; once depleted, the cells consumed fructose. This pattern of sugar consumption has been reported previously (Caspeta et al., 2005; Pérez, 2019).

The kinetic parameters determined for SF-WT and SF-23 at the flask level guided the selection of the SF-23 line for scale-up. The maximum biomass obtained at flask level (14.36 g/L DW) was higher than that obtained in the bioreactor (12.8 g/L DW); this reduction in cell growth in the bioreactor could be due to the presence of small cell aggregates, which caused mixing problems. Differences in the conditions of both scales, such as agitation and system geometry, could also have played a role. However, the airlift bioreactor operating conditions were favorable for cell growth, as it has been reported for other species (Trejo and Rodríguez, 2007).

Excessive use of anthelmintics to control parasitic nematodes in sheep is leading to drug resistance. On the other hand, macrocyclic lactones (avermectins, milbemycins, and spinosins) have shown toxicity and adverse effects on *Scarabaeus viettei* and *S. laticollis*, arthropod species whose presence is beneficial to natural and livestock grazing environments (Jacobs and Scholtz, 2015). To avoid these problems, the use of transformed plant cells has been proposed as an alternative to control gastrointestinal nematodes. Despite the promising results obtained with alternative drugs, to date, there is no effective and safe natural anti-parasitic product on the market. The use of transgenic plants to control gastrointestinal nematodes in ruminants could allow us to design cost-effective parasite control strategies and to offer safer products for livestock caretakers.

In this regard, *C. papaya* has drawn considerable interest for its anthelmintic activity. Indeed, papaya extracts effectively inhibited *Trichostrongylus colubriformis* egg hatching and larval migration (Hounzangbe-Adote et al., 2005). The anthelmintic capacity of papaya has been linked to its content of proteolytic enzymes such as papain, chymopapain, caricain, and glycyl endopeptidase (Luoga et al., 2015; Turuj and Yash, 2015). Benzyl isothiocyanate has been identified as the predominant or sole anthelmintic agent in papaya seed extracts (Kermanshai et al., 2001). These active components in papaya latex effectively reduced *H. contortus* burden by 98% when orally administered to sheep (Buttle et al., 2011). The stability of papaya cysteine proteinases in the latex has been recently reported (Mansur et al., 2021).

The nematocidal activity shown by the biomass of the SF-23 transgenic line of *C. papaya* against L_4_ larvae of the parasitic nematode *H. contortus* indicates a direct action on internal organs by hydrolysis of specific peptide bonds in proteins, and a loss of cuticular integrity. Biomass from the transgenic line SF-23 (1 mg/ml) sampled after 14 days of culture showed similar efficacy to that of Ivermectin (5 mg/ml). In contrast, the maximum mortality rate for SF-WT biomass after 14 days of culture was only 7.03%. All previously reported nematocidal properties of papaya were limited to latex and seeds, and this is the first report on the nematocidal activity of transformed *C. papaya* suspension cells. The increased activity of the SF-23 transgenic with respect to SF-WT could be a consequence of the insertion of the gene coding for the peptide KETc7, which clearly modified the protein profile of cells, as shown in **Figure 4**.

As shown in **Figure 5A**, mortality rate reached a maximum when larvae were treated with extracts sampled after 4 days of culture, and this rate remained virtually unchanged when extracts were sampled at days 10 and 14 of culture. It is feasible that the levels of the most active metabolites, such as papain, reach a maximum on day 4 of culture.

As a first approach to elucidate the modifications induced by the KETc7 gene insertion in the protein content of papaya callus, 2D electrophoresis was performed. A lower number of protein spots were found in the callus cell line CF-23 with respect to wild-type CF-WT, indicating a different protein content (**Figure 3**). Similar findings have been reported in other genetically modified plants (Barbosa et al., 2012; Tan et al., 2017; de Campos et al., 2020). The CF-23 transgenic line produced two spots (41 and 50) whose MW and IP values resemble chymopapain, the main cysteine proteinase in papaya. Interestingly, both spots are absent in wild-type callus cells. It is likely that these differences are also found between the SF-23 and SF-WT lines. Thus, cysteine proteinases could be involved in the higher nematocidal activity of SF-23. Considering the relevance of this finding, further experiments will be conducted to evaluate this possibility.

When comparing the protein profiles of CF-WT and CF-23, a difference in the number of spots was found between both cell lines. CF-23 expresses a lower number of spots (proteins), which could be related to its higher growth capacity at the expense of producing fewer proteins, as this could imply a higher production of other components with nematocidal capacity. This could be either because the transgene was inserted into sites that affect protein expression or, conversely, that it was inserted into sites that induce the expression of enzymes that degrade other proteins. Further studies are needed to explore this possibility, and it would be important to identify which proteins are differentially expressed in the two cell lines.

In summary, these results lay the groundwork for evaluating the potential of *C. papaya* suspension cell lines to control *H. contortus* infection while avoiding stationary or ontogenetic changes in the chemical composition of papaya cultivars (Selvaraj et al., 1982). Although these results are interesting, *in vivo* experiments are crucial to support the potential of these products as natural eco-friendly antiparasitic medications, and to explore the mechanisms that mediate their nematocidal activity.

## Data availability statement

The original contributions presented in the study are included in the article/**Supplementary Material**. Further inquiries can be directed to the corresponding authors.

## Author contributions

AOC and MV conceived and designed experiments for *in vitro* cell cultivation, analyzed results, and wrote the paper. AP and CG performed cell suspension cultivation. MH produced the transgenic and wild-type calluses and designed and performed the PR analysis. DA-N and RB designed and performed the protein expression analysis. LA and ML performed *in vitro* nematocidal bioassays. ES and GF accomplished the analysis of results and discussion and reviewed the complete final manuscript. All authors contributed to the article and approved the submitted version.

## Funding

This work was supported by the Institutional program “Programa de Investigación para el Desarrollo y la Optimización de Vacunas, Inmunomoduladores y Métodos Diagnósticos del Instituto de Investigaciones Biomédicas” (PROVACADI), UNAM and CONACYT 222714 awarded to MLV.

## Acknowledgments

We thank Pérez, R. C, Jiménez, C. A., and Campos, C. for providing information from their thesis works, L.I. Omar Rangel for his technical assistance (IIBO, UNAM), and Arianne Melisa Cristino Miranda for her technical assistance.

## Conflict of interest

The authors declare that the research was conducted in the absence of any commercial or financial relationships that could be construed as a potential conflict of interest.

## Publisher’s note

All claims expressed in this article are solely those of the authors and do not necessarily represent those of their affiliated organizations, or those of the publisher, the editors and the reviewers. Any product that may be evaluated in this article, or claim that may be made by its manufacturer, is not guaranteed or endorsed by the publisher.

## Abbreviations

*C. papaya, Carica papaya; H. contortus, Haemonchus contortus;* CF-23, transformed callus line; CF-WT, non-transformed callus line; SF-23, transformed suspension cultures; SF-WT, untransformed suspension cultures; FW, fresh weight; DW, dry weight; HPLC, high-performance liquid chromatography.
