# Supplementary material for: Improvement of cell suspension cultures of transformed and untransformed Carica papaya cell lines, towards the development of an antiparasitic product against the gastrointestinal nematode Haemonchus contortus
Source: Front Cell Infect Microbiol. 2022 Sep 8;12:958741. doi: 10.3389/fcimb.2022.958741 (PMC9493254; doi:10.3389/fcimb.2022.958741)
Supplement: Supplementary file 1 [file Table_1.pdf]

Supplementary Table 1

Wild-type			CF-23 clone		
**No. Spot**	**MW (kDa)**	**IP**	**No. Spot**	**MW (kDa)**	**IP**
1	100	6.5	1	85	7
2	90.89	5.1	2	78.5	5
3	90.89	4.5	3	50	4.5
4	90.89	3.3	4	55	5.1
5	87.48	4.5	5	55	5.3
6	100	5.6	6	47.44	5.4
7	100	5.7	7	47.08	5.57
8	90.89	6.05	8	46.36	5.6
9	87.48	5.8	9	45.64	5.85
10	92.025	5.6	10	47.44	5.8
11	77.27	5.8	11	43.48	29.56
12	77.27	6.05	12	41.32	4.8
13	78.5	4.9	13	35.96	5.3
14	78.5	5	14	35.9	5.35
15	78.5	5.1	15	41.32	5.38
16	62	4.2	16	41.32	5.5
17	62	4.4	17	41.32	5.6
18	62	6.5	18	41.32	5.6
19	62	4.7	19	41.32	5.7
20	62	5.65	20	41.32	5.8
21	62	5.8	21	41.32	5.9
22	60	5	22	41.32	6
23	60	5.1	23	37.72	6.2
24	60	4.7	24	37.72	5.1
25	60	5.4	25	37.72	5.25
26	60	5.5	26	30.61	5.4
27	56	3.8	27	32.26	4.85
28	58	4.5	28	32.26	5.25
29	57	5.4	29	28.3	5.25
30	57	5.5	30	30.94	5.25
31	57	5.55	31	43.47	5.5
32	56	5.6	32	30.94	5.6
33	56	5.7	33	30.94	5.6
34	55	5.8	34	28.3	5.8
35	53	6.15	35	29.62	5.8
36	54	6.15	36	30.94	5.85
37	54	6.3	37	29.29	6.2
38	54	6.25	38	26.65	6
39	53	6.75	39	28.3	5.9

40	53	5.2
41	53	5.3
42	54.5	5.4
43	54.5	5.5
44	54.5	5.5
45	54.5	5.6
46	54.5	5.7
47	54.5	5.9
48	54.5	6.05
49	51	4.85
50	51	5.1
51	51	5.3
52	52	5.6
53	52.5	5.8
54	52.5	5.9
55	52.5	5.8
56	52.5	5.85
57	37.86	8.6
58	48.18	5.8
59	49.04	6
60	47.32	6.1
61	44.2	5
62	44.2	5.1
63	44.2	5.3
64	44.2	5.4
65	43.88	5.5
66	43.88	5.55
67	43.88	5.57
68	43.02	5.7
69	43.02	5.8
70	43.02	5.9
71	43.02	6.1
72	43.88	6.4
73	43.88	6.7
74	42.16	6.7
75	41.3	5.38
76	41.3	5.8
77	41.5	5.7
78	41.3	5.8
79	41.3	5.8
80	41.3	5.8
81	39.58	6.03
82	39.58	6.2
83	41.5	6.95
84	38.72	6.95

40	26.65	6.2
41	24.33	6.9
42	30.94	6.4
43	28.79	7.5
44	26.65	7.5
45	27.64	8.5
46	26.98	8.6
47	25	8.45
48	25	8.6
49	23.57	5.9
50	23.14	8.3

85	37.86	6.95
86	41.5	7.2
87	36.73	7.9
88	39.58	4.8
89	37.42	4.15
90	35.12	3.65
91	35.12	5.2
92	36.73	5.3
93	36.27	5.5
94	36.73	5.6
95	36.73	5.8
96	33.28	5.95
97	33.28	5.6
98	30.98	7.15
99	30.06	3.7
100	29.6	4.3
101	29.6	3.6
102	29.37	6
103	28.68	6
104	27.07	6
105	27.07	5.4
106	28.6	5.5
107	25	5.8
108	25	5.4
109	24	5.5

2
